# A Confidence-Aware Hybrid Vision–Language Framework for Food Recognition and Nutritional Monitoring

**DOI:** 10.3390/nu18152449

**Published:** 2026-07-27

**Authors:** Furkan Göz, Muhammad Jamil, Adnan Kavak, Sema Bayraktar, Ali Can Doğru, Gautam Srivastava, Hossein Fotouhi

**Affiliations:** 1 Department of Computer Engineering, Kocaeli University, 41001 İzmit, Türkiye; furkan.goz@kocaeli.edu.tr (F.G.); jamil138.amin@gmail.com (M.J.); ali.can.dgru10@gmail.com (A.C.D.); 2 Wireless Information and Intelligent Systems (WINS) Research Center, Kocaeli University, 41001 İzmit, Türkiye; bayraktar.sema41@gmail.com; 3 Sogang University, Seoul 04107, Republic of Korea; 4 Research Center for Interneural Computing, China Medical University, Taichung 40402, Taiwan; 5 Centre for Research Impact & Outcome, Chitkara University Institute of Engineering and Technology, Chitkara University, Rajpura 140401, Punjab, India; 6 Department of Computer Science and Engineering, Mälardalen University (MDU), 72123 Västerås, Sweden

**Keywords:** digital nutrition, food image recognition, nutrition estimation, multimodal large language models, confidence-aware routing, personalized dietary assessment, mobile health

## Abstract

**Background/Objectives:** The objective evaluation of regional dietary intake remains a core challenge in personalized health management due to complex plate presentations and a lack of culturally specific dataset benchmarks. **Methods:** This study introduces a confidence-aware hybrid vision–language framework engineered for traditional Turkish food recognition and structured nutritional assessment. **Results:** We curate a balanced dataset containing 14,711 verified images spanning 40 representative Turkish culinary classes to train and evaluate seven deep learning architectures. Among the visual models, EfficientNet V2-L achieved the highest standalone performance with an accuracy of 93.47%, 0.92 macro-precision, 0.92 macro-recall, and a 0.92 F1 score. To overcome visual ambiguity and automate content analysis, a confidence-aware routing strategy escalates uncertain predictions (τ<0.70) or user-rejected classifications to the Google Gemini 2.5 Flash multimodal large language model (MLLM). **Conclusions:** This hybrid paradigm yields a combined classification accuracy of 95.50% while validating portion weight estimations within a mean absolute error (MAE) of 18.42 g and total energy within 36.75 kcal. Fully realized as a cross-platform Flutter mobile application, the end-to-end pipeline demonstrates localized plate detection, adaptive portion analysis, and structured nutrient tracking, providing a scalable design for consumer-facing digital nutrition platforms.

## 1. Introduction

Diet-related health conditions, such as obesity, diabetes, and cardiovascular disorders, require reliable diet monitoring approaches to support disease prevention and health management [1,2]. To address this, computer vision techniques are employed to automatically identify meals from source images for personalized lifestyle management and healthcare-related nutrition tracking. Several studies have demonstrated the utility of food image recognition in addressing health challenges [3,4]. However, recognition of food categories alone is not sufficient for comprehensive dietary monitoring. Reliable assessment requires portion size estimation and nutritional analysis tasks that remain challenging for visual systems [5,6].

Image classification techniques enable decision support systems to identify food categories without user intervention. These approaches have been effectively applied to nutrient estimation, food quality analysis, and the classification of culturally diverse dishes using Convolutional Neural Networks (CNNs) and transformer-based architectures [7]. Food classification remains a laborious task due to wide variations in food presentation, ingredients, cooking methods, and inter-class visual similarities [8].

Most studies in the literature have focused on Western cuisine, using standard benchmarks such as Food-101 [9]. These datasets have been widely used [10] but lack diversity, which is a major constraint for regional cuisines. Generating datasets for a specific area is imperative for enhancing performance [11]. The cross-dataset applicability of pre-trained models has recently been demonstrated across various domains [12,13,14].

In this research, we address these gaps by curating a well-balanced set of images of Turkish cuisine and evaluating the performance of state-of-the-art deep learning models. A special dataset comprising 40 classes of representative traditional dishes was prepared as a reference for comparing different architectures. We chose these models because of their extensive use in food recognition tasks [15]. Moreover, to augment the visual classification with the capability for portion size estimation and nutritional inference, the present work incorporates a large language model (LLM). The LLM further enhances the visual models by enabling structured extraction of nutritional information and offering context-aware support. The entire framework was implemented as a mobile application developed using Flutter to test its feasibility for real-world dietary tracking.

The central methodological contribution of the proposed framework is a confidence-aware multimodal routing strategy for culturally specific food analysis. The visual classifier first generates the predicted food category and its associated confidence score. Predictions with sufficient confidence are processed directly, whereas uncertain predictions or predictions rejected by the user are selectively forwarded to the vision–language model for multimodal reassessment and structured nutritional interpretation. This selective escalation introduces uncertainty handling into the inference workflow while avoiding unnecessary use of the multimodal model for every input image.

The principal contributions of this study are presented below:

- We introduced a comprehensive image dataset representing Turkish cuisine to address the scarcity of culturally specific resources in the current literature.
- We evaluated six CNN architectures and a Vision Transformer under a common experimental protocol to compare their performance on regional food recognition.
- We introduced a confidence-aware multimodal routing strategy in which classifier confidence and user feedback determine whether a prediction is retained or selectively escalated to a vision–language model for additional interpretation and structured nutritional analysis.
- We developed an end-to-end mobile application to demonstrate the system’s applicability for daily dietary monitoring.

## 2. Related Work

Traditional approaches, such as manual feature extraction and the resulting poor generalization, have limited the use of food image classification, thereby making the CNN-based deep learning approach dominant. Key challenges include running on resource-limited platforms, performing fine-grained classification (i.e., distinguishing visually similar dishes), and the lack of large, balanced datasets for the various cuisines. Recent research has focused on model architectures, learning strategies and special techniques to address such issues [16,17].

Food item recognition based on images is difficult due to the deformable nature of food items and their semantic complexity, high intra-class variation, and low inter-class differences. Kiourt et al. (2020) presented a comparative analysis of various deep learning solutions, emphasizing the trade-off between model performance and practical requirements as the model with the highest accuracy is not necessarily the best choice in terms of efficiency and computational cost [18]. More recently, Deng et al. (2024) [19] presented a high-level model built on the ConvNeXt (CXT) architecture that incorporates a hybrid attention (HA) mechanism and a multi-stage local fusion (MSLF) module to tackle these complexities. This method was used to improve feature extraction, and the highest accuracy rates were 91.12%, 82.86% and 92.50% for the Food-101, ChineseFoodNet and Roushi60 datasets, respectively [19]. In addition to classification, real-world dietary uses require precise portion estimation and structured nutritional information, which traditional vision-based methods fail to provide. The literature is lacking in these respects, especially in the context of culturally diverse cuisines.

One basic method in this field is to design lightweight model architectures for use in mobile and embedded systems. To reduce computational cost, the MSNet deep learning architecture has been proposed, in which the M and S blocks optimize information flow. Quantized variants of MSNet-Lite are suitable for mobile applications [20]. MobileNetV2 transfer learning also showed considerable improvements in accuracy and efficiency compared to a self-developed CNN model in a nutrition monitoring system [21]. These studies emphasize the need for and feasibility of lightweight models in resource-constrained settings.

One investigation was carried out using transfer learning with a small dataset of 10 classes of food images in Indonesia with an accuracy reaching 87.25% by employing an EfficientNet V2 model and focusing on data augmentation and Grad-CAM visualization [22]. Similarly, fine-tuning the Xception architecture on the Fast Food V2 dataset achieved 95.14% accuracy, precision, and recall [23]. A comparative evaluation on the Food-11 dataset indicated that EfficientNet B7 offered high trainability while ResNet50 provided more balanced generalization. In addition, restructuring of VGG19 with layer freezing, data augmentation, dropout, and early stopping successfully suppressed overfitting with 83% accuracy [24].

There are studies focusing on regional cuisine and large-scale datasets. For example, the MedGRFood dataset, comprising 132 classes and 42,880 images of Mediterranean cuisine, was developed for food recognition tasks. Using transfer learning with the EfficientNet B2 architecture, a Top-1 accuracy of 83.4% was achieved [25]. In a similar work, a 150-class Korean food dataset comprising approximately 150,000 images—augmented to approximately 527,000—was used to fine-tune ResNet V2, Inception-ResNet V2, NasNetLarge, and MobileNetV2. Inception-ResNet V2 obtained the highest overall accuracy, while certain augmentation methods were found to affect performance [26]. Challenges associated with fine-grained classification have driven the development of the LMB-Net model, which integrates a Laplacian pyramid-based texture detection layer and multi-scale bilinear pooling modules. This approach achieved Top-1 accuracies of 85% and 93% on the ChineseFoodNet and VireoFood172 datasets, respectively [27].

For the applications related to Turkish cuisine, the TurkishFoods-15 dataset was created with 15 classes and 7500 images. The images were scraped from Google Images and manually cleaned. The dataset was then combined with different classes to form a baseline set of 113 classes and 113,000 images. Fine-tuning the final layer of the Inception V3 model on this dataset achieved an accuracy of 68.2% [28]. Then, EfficientNet V2-M, ResNet101 and VGG16 were fine-tuned on the TurkishFoods-15 dataset. Moreover, a hybrid system was designed based on features extracted from the Vision Transformer (ViT) and a Random Forest (RF) Classifier. Based on overall performance [29], the ViT-based approach outperforms CNN models.

In addition to visual classification, research has focused on estimating portion size [30] and deriving nutritional information from food images [31]. One of the most recent reviews focused on automatic portion-size and carbohydrate-estimation mobile apps and their challenges, such as the need to make simplified assumptions, small datasets and low generalizability across a broad spectrum of foods. Meanwhile, there is growing interest in vision–language and multimodal models for food-related applications such as recipe retrieval, food question answering [32], and nutrition guidance [33,34]. However, systematic evaluations remain limited, particularly for deployment on mobile devices and across cuisines from culturally diverse backgrounds. Table 1 provides a summary of related studies.

Because the reported studies use different datasets, class distributions, and evaluation protocols, their results are presented for contextual comparison and should not be interpreted as a direct ranking against the present study.

## 3. Methodology

**Figure 1:**
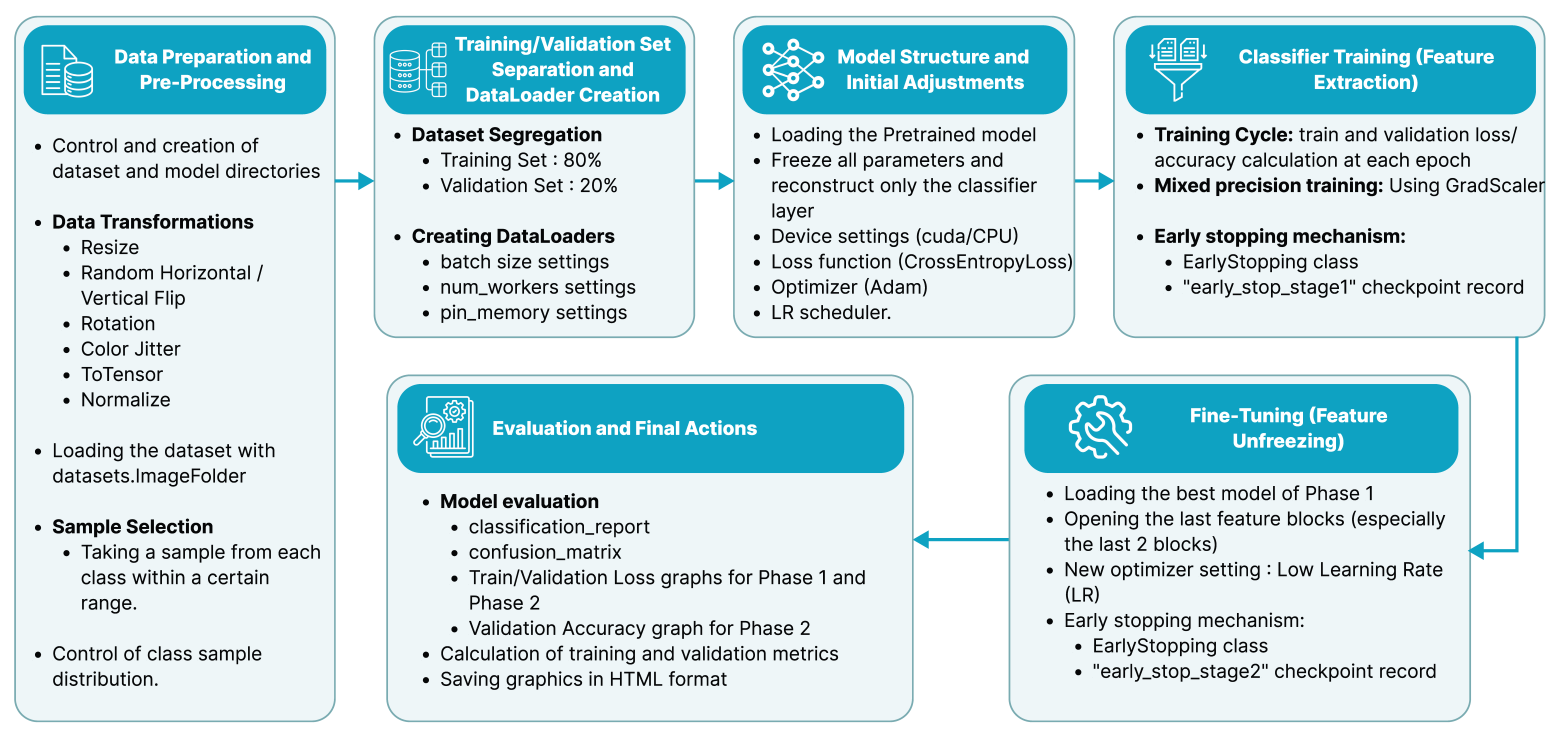
Flowchart visually summarizing the methodology.

The proposed framework is presented in this section, starting with the curation of the custom Turkish cuisine dataset and the preprocessing techniques used to improve model generalization. Next, we analyze the performance of the EfficientNet, Inception, Inception-ResNet, Vision Transformer, and ResNet50 models that have been adapted using transfer learning. The following defines the metrics used to evaluate the application’s performance. The integration of a MLLM for nutritional analysis and the deployment of the system on a mobile platform are presented. The entire CNN-based classification pipeline is shown in Figure 1. The pipeline has two stages: a feature extraction stage with the backbone parameters fixed and a fine-tuning stage with some of the layers unfrozen to learn domain-specific features.

### 3.1. Dataset Preparation

**Figure 2:**
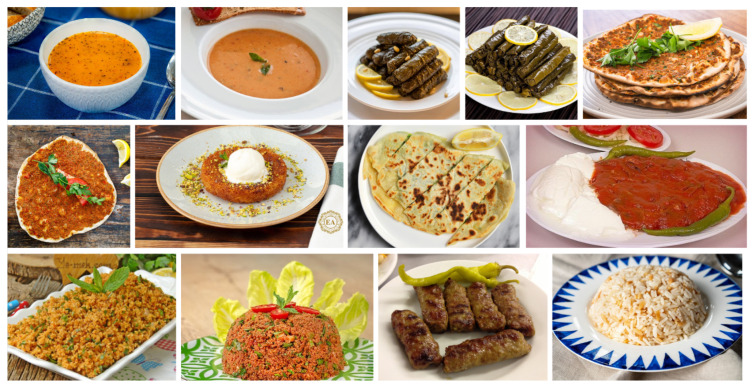
Representative examples from the Turkish food image dataset.

A food-recognition dataset containing 40 categories of traditional Turkish foods was developed for this study. Images were collected from publicly accessible web sources and processed through a multi-stage curation procedure. Representative examples from the dataset are presented in Figure 2.

In the initial collection stage, 22,635 food images were obtained across the 40 categories, denoted by $N_{\mathrm{raw}}$. Before class balancing and dataset partitioning, a duplicate-control and quality-screening procedure was conducted to reduce the possibility of data leakage. Exact duplicate files were identified using SHA-256 cryptographic hashes. Near-duplicate images, including resized, cropped, compressed, or slightly modified versions of the same source image, were subsequently screened using perceptual hashing. Image pairs with a perceptual-hash Hamming distance of five or less were manually inspected. Confirmed duplicate groups were removed before dataset partitioning, and images originating from the same source record or belonging to the same near-duplicate group were restricted to a single partition.

In total, 1842 exact duplicates and 1736 confirmed near-duplicates were removed. An additional 4346 images were excluded because of blurring, low resolution, incorrect labeling, irrelevant content, other visual-quality problems, and class balancing. Therefore, a total of 7924 images were excluded from the original collection.

Because the number of available images varied across the food categories, a class-balancing procedure was applied to limit the degree of class imbalance while retaining sufficient intra-class variation. Let $n_{c}$ denote the number of available images in class *c*. The size of the smallest class is then given by:

(1) $$n_{\min}=\min_{c}n_{c}$$

A tolerance parameter of δ=50 was used to determine the number of images retained for each category:

(2) $$n_{\mathrm{cap}}=n_{\min}+\delta$$

The number of images retained for each class was then defined as:

(3) $$n_{c}^{\prime }=\min\left(n_{c},n_{\mathrm{cap}}\right)$$

The retained samples $\left\{n_{c}^{\prime }\right\}$ formed the final dataset. This procedure limited the difference between the smallest and largest retained classes to at most δ=50 images, thereby reducing class imbalance while preserving available visual diversity.

After duplicate removal, quality screening, manual verification, and class balancing, the final dataset contained $N_{\mathrm{final}}=14,711$ images. The cleaned dataset was divided into a training set containing $N_{\mathrm{train}}=11,768$ images and a test set containing $N_{\mathrm{test}}=2943$ images, corresponding approximately to an 80:20 split.

The dataset partitioning was performed only after duplicate-group verification, ensuring that exact or near-duplicate variants were not distributed across both the training and test sets.

#### Independent External Evaluation Set

To assess the generalizability of the selected model beyond the web-sourced benchmark, an independent external test set containing $N_{\mathrm{ext}}=105$ images was created after model training and selection. The images were captured using smartphones in home, restaurant, and cafeteria environments and included variations in illumination, viewing angle, background complexity, partial occlusion, food presentation, and the number of plates. None of these images, their source variants, or their near-duplicates were included in the training or validation partitions. The external set was used only for final evaluation and was not used for hyperparameter tuning or model selection. This evaluation examines robustness to different acquisition conditions within the 40 Turkish food categories considered in this study.

The evaluation focused on plate-level recognition of individual dishes. Images captured in homemade, restaurant, and cafeteria environments included variations in illumination, viewing angle, background complexity, and partial occlusion. When multiple plates were present, each detected plate was selected and analyzed separately; mixed-food recognition within a single plate was not evaluated.

### 3.2. Data Pre-Processing

The training set was used with a pre-processing and data augmentation pipeline to improve the model’s generalization and avoid overfitting. Only deterministic resizing and normalization were used for the test and validation sets; all transformations were applied online during training. This separation prevents stochastic augmentations from affecting the evaluation results, and reported performance is a true measure of generalization.

#### 3.2.1. Resizing

All images were resized to the fixed spatial inputs of each model to accommodate the different resolutions of the raw images and ensure consistent spatial inputs across models. Specifically, EfficientNet V2-L and EfficientNet V2-XL were scaled to 480×480 pixels; Inception V4 and Inception-ResNet V2 were scaled to 299×299 pixels; ResNet50 and the ViT-Huge-Patch14-224 model were scaled to 224×224 pixels; and EfficientNet B7 was scaled to 600×600 pixels. The model-specific scaling method is compatible with pre-trained weights. It does not change the model’s architecture, keeping the spatial resolution the same throughout training and inference.

#### 3.2.2. Geometric Augmentations

Random horizontal flipping was applied with 50% probability and vertical flipping with 20% probability to introduce diversity in the effective training data and make it more robust to common geometric variations. Furthermore, images were randomly rotated over the range $\left[-15^{\circ },+15^{\circ }\right]$ to simulate moderate variations in viewpoint and camera angle. These geometric transformations promote the learning of rotation- and orientation-invariant feature representations, especially for food images in unconstrained mobile environments.

#### 3.2.3. Photometric Augmentations

Photometric perturbations were added to increase the robustness of the data to illumination and sensor variability by random changes in brightness, contrast, saturation and hue. The color-based augmentations provide greater robustness to changes in lighting, camera and environment, leading to more robust feature extraction across a variety of acquisition scenarios.

#### 3.2.4. Normalization

The image tensors were resized and augmented before being normalized per channel using mean and standard deviation values calculated from the ImageNet dataset. This normalization can eliminate scale differences between channels, stabilize optimization, and be compatible with the parameters of the pretrained backbone. Consequently, better convergence behavior is achieved, and training stability is increased for all evaluated architectures.

### 3.3. Model Architectures

Seven pretrained architectures were evaluated to compare different feature-extraction strategies for fine-grained Turkish food recognition. The selected models represent residual learning, multi-scale convolution, compound scaling, and transformer-based self-attention. These architectures were evaluated with the same dataset partition, training procedure, and performance criteria to ensure a consistent comparison.

#### 3.3.1. EfficientNet Models

EfficientNet B7, EfficientNet V2-L, and EfficientNet V2-XL were included because they provide high representational capacity through compound scaling of network depth, width, and input resolution [35,36]. EfficientNet B7 uses MBConv blocks with squeeze-and-excitation modules, whereas the EfficientNet V2 variants combine fused MBConv blocks in the earlier stages with standard MBConv blocks in deeper stages. EfficientNet V2-L and V2-XL were evaluated to examine whether the increased capacity of the larger variant produced meaningful improvements for visually similar food categories.

#### 3.3.2. Inception V4 and Inception-ResNet V2

Inception V4 uses parallel convolutional paths with different kernel sizes to capture visual patterns at multiple spatial scales [37]. Inception-ResNet V2 combines this multi-scale processing with residual connections to improve gradient propagation and optimization. These models were included because food images often contain substantial variation in shape, texture, presentation, and viewing scale.

#### 3.3.3. ResNet50

ResNet50 employs residual shortcut connections to support the optimization of deep convolutional networks [38]. Its bottleneck blocks combine 1×1 and 3×3 convolutions to learn hierarchical visual features. ResNet50 was included as a widely used residual-learning baseline for comparison with the larger EfficientNet, Inception, and transformer architectures.

#### 3.3.4. ViT-Huge-Patch14-224

ViT-Huge-Patch14-224 applies the Vision Transformer architecture to image classification [39]. A 224×224 input image is divided into non-overlapping 14×14 patches, which are embedded as a sequence of visual tokens. Multi-head self-attention is then used to model both local patch-level information and long-range relationships across the image. This model was included to compare transformer-based feature learning with convolutional architectures for the food-recognition task.

For all architectures, the original classification layer was replaced with a 40-class output layer corresponding to the food categories in the proposed dataset. The models were initialized using ImageNet-pretrained weights and subsequently adapted using the same two-stage transfer-learning procedure described in Section 3.4.

### 3.4. Model Training

In this study, all deep learning models were initialized using pretrained weights obtained from training on the ImageNet dataset. The final classification layers of each architecture were restructured to match the number of classes in the proposed dataset. A two-stage transfer learning strategy was adopted in order to leverage pretrained representations while adapting the models to the target food recognition task. In the first training stage, only the classifier head was optimized, while the backbone feature extraction layers were frozen. This configuration ensures that the high-level visual representations learned from ImageNet are preserved, while allowing the newly initialized classification layer to adapt to the domain-specific class distribution.

In this phase, the optimization was performed using the Adam optimizer, and early stopping was dynamically implemented based on the validation loss to halt training. The number of epochs, the learning rate, and the batch size were chosen based on the memory capacity and performance of the available GPU hardware. A fine-tuning procedure was used in the second stage to improve classification performance further. In particular, the last two blocks of the backbone network were unfrozen, and a subset of the deeper feature representations was adapted to the target domain. This stage was initialized with the best-performing model weights from the first stage.

To avoid large weight updates that could affect the learned representations, one-tenth of the initial learning rate was used during fine-tuning. This stage was also aided by implementing early stopping and learning-rate scheduling via the ReduceLROnPlateau strategy.

In all experiments, the number of training epochs was 25 and an early stopping criterion was used to prevent overfitting: training was stopped when validation loss failed to improve for 5 consecutive epochs, maximizing training efficiency. To ensure stable and efficient convergence across various network architectures, the Adam optimizer was used throughout. The batch size for each model was adjusted based on model complexity and GPU memory.

For ResNet50, EfficientNet V2-L, Inception V4, and Inception-ResNet V2, a batch size of 256 was used. For the larger and more memory-intensive models (EfficientNet B7, EfficientNet V2-XL, and ViT-Huge), a batch size of 64 was used. This setup will optimize GPU memory usage and provide a regularization effect for larger models by using smaller batch sizes. Eight parallel workers were used for all models except the ResNet50 model, which used four workers for data loading. The following training parameters were selected as standardized parameters for fair comparison between the architectures and optimal use of computational resources.

### 3.5. Statistical Analysis

Ninety-five percent confidence intervals for accuracy, macro-precision, macro-recall, and macro-F1 score were estimated using 1000 bootstrap resamples of the test set. Because all models were evaluated on the same images, Cochran’s *Q* test was first applied to examine the overall difference in prediction correctness among the models. When the overall test was significant, pairwise McNemar tests were conducted, and the resulting *p*-values were adjusted using the Holm procedure. Statistical significance was defined as an adjusted p<0.05.

All architectures were evaluated on the same held-out test set to enable paired statistical comparison under identical conditions; repeated cross-validation was not performed because of the computational cost of retraining all seven models.

### 3.6. Integration of a Multimodal LLM

Google Gemini 2.5 Flash was integrated through the Generative Language API to extend the visual-recognition pipeline beyond food classification. Predefined prompt templates and a fixed JSON schema were used to improve output consistency and facilitate downstream processing. The model was not employed as an independent replacement for the food classifier; instead, it supported multimodal reassessment, portion estimation, nutritional-data structuring, text-based food logging, and profile-conditioned nutritional guidance.

The prompt required structured outputs for food identity, portion weight, energy, carbohydrate, protein, and fat, while instructing the model to express uncertainty and avoid diagnostic, medication, or treatment advice.

The vision–language model estimates portion weight from a food image or textual description and generates structured values for energy, carbohydrate, protein, and fat. The generated output is parsed into a predefined nutritional-record format before being stored by the application. Users may also provide textual entries, such as “150 g of chicken breast” or “one plate of Menemen,” which are converted into structured food and nutrient records.

In this study, personalization refers to profile-conditioned nutritional support based on user characteristics, dietary preferences, calorie targets, and recorded food history. It does not represent clinically validated individualized treatment or demonstrate improvements in dietary adherence or health outcomes.

The application was developed as a research and general nutritional-support prototype rather than a diagnostic or clinical decision-support system. Only limited user-provided profile information is used to condition nutritional suggestions, and the model is instructed not to provide diagnoses, medication changes, or treatment decisions. Any future healthcare deployment will require formal privacy and security assessment, demographic bias evaluation, clinical validation, and jurisdiction-specific regulatory review.

### 3.7. End-to-End Pipeline Evaluation

The complete workflow was evaluated on $N_{e2e}=105$ meal images. The evaluated stages included plate detection, food recognition, portion estimation, nutritional estimation, and nutritional-guidance generation. Plate detection was considered successful when the relevant plate was correctly localized, while food recognition required the predicted class to match the reference label. Portion and nutritional estimates were considered acceptable when their absolute percentage errors were within 20% of the reference values. Generated guidance was considered successful when it was factually acceptable, consistent with the user profile, and contained no unsafe recommendation. For image *i*, the end-to-end (E2E) success indicator is defined as:

(4) $$S_{i}=D_{i}C_{i}P_{i}N_{i}A_{i}$$

where $D_{i}$, $C_{i}$, $P_{i}$, $N_{i}$, and $A_{i}$ represent successful plate detection, food classification, portion estimation, nutritional estimation, and advice generation, respectively. Each variable takes a value of 1 for success and 0 otherwise. The complete-pipeline success rate was calculated as:

(5) $$E2\mathrm{ESuccess}=\frac{1}{N_{e2e}}\sum_{i=1}^{N_{e2e}}S_{i}\times 100$$

#### 3.7.1. Reference-Based Validation of Nutritional Estimation

The portion-size and nutritional-estimation functions were evaluated using a controlled reference set containing $N_{\mathrm{nutr}}=105$ meal images. The reference portion of each food item was measured using a digital kitchen scale. Reference values for energy, carbohydrate, protein, and fat were calculated from the measured portion weight using a validated food-composition database and were subsequently checked for consistency. For each meal image, the vision–language model estimated portion weight and the corresponding nutritional composition. Table 2 summarizes the reference sources, units, and evaluation criteria used for each variable.

For a reference value $y_{i}$ and its corresponding estimate ${\hat{y}}_{i}$, the mean absolute error (MAE) and root mean squared error (RMSE) were calculated as:

(6) $$\mathrm{MAE}=\frac{1}{N}\sum_{i=1}^{N}\left|y_{i}-{\hat{y}}_{i}\right|$$

(7) $$\mathrm{RMSE}=\sqrt{\frac{1}{N}\sum_{i=1}^{N}{\left(y_{i}-{\hat{y}}_{i}\right)}^{2}}$$

The mean absolute percentage error (MAPE) was calculated for samples with nonzero reference values:

(8) $$\mathrm{MAPE}=\frac{100}{N_{+}}\sum_{i\in I_{+}}\left|\frac{y_{i}-{\hat{y}}_{i}}{y_{i}}\right|$$

where $I_{+}=\left\{i:y_{i}>0\right\}$ and $N_{+}$ is the number of samples with nonzero reference values. The metrics were calculated separately for portion weight, energy, carbohydrate, protein, and fat.

#### 3.7.2. Factuality, Consistency, and Safety Evaluation

The factual reliability and safety of the vision–language model were evaluated using a predefined set of $N_{\mathrm{safe}}=100$ nutrition-related scenarios. The scenarios included general nutritional questions, portion-related queries, dietary restrictions, allergy-related requests, weight-management questions, and prompts requesting medical diagnosis or treatment advice. Each generated response was evaluated according to four criteria: factual correctness, consistency with the supplied user profile, presence of unsupported or hallucinated claims, and the occurrence of potentially unsafe nutritional recommendations. Responses were compared with reference nutritional information and independently reviewed using a predefined evaluation form.

To assess output consistency, each scenario was submitted three times using identical input conditions. A response was considered inconsistent when repeated outputs contained materially different nutritional values, recommendations, or safety guidance. The factual accuracy, hallucination, inconsistency, and safety-compliance rates were calculated as:

(9) $$\mathrm{Factual}\;\mathrm{Accuracy}=\frac{N_{\mathrm{factually}\;\mathrm{correct}}}{N_{\mathrm{total}}}\times 100$$

(10) $$\mathrm{Hallucination}\;\mathrm{Rate}=\frac{N_{\mathrm{unsupported}}}{N_{\mathrm{total}}}\times 100$$

(11) $$\mathrm{Inconsistency}\;\mathrm{Rate}=\frac{N_{\mathrm{inconsistent}}}{N_{\mathrm{total}}}\times 100$$

and

(12) $$\mathrm{Safety}\;\mathrm{Compliance}=\frac{N_{\mathrm{safe}}}{N_{\mathrm{total}}}\times 100$$

The model was instructed not to provide medical diagnoses, medication changes, or treatment decisions. High-risk prompts were expected to produce a cautionary response and recommend consultation with an appropriately qualified healthcare professional.

#### 3.7.3. Confidence-Aware Multimodal Routing

The vision–language model was integrated through a confidence-aware routing mechanism rather than being applied to every input image. Let $p_{\max}$ denote the highest class probability produced by the EfficientNet V2-L classifier, and let τ denote the routing threshold. The final inference route is defined as:

(13) $$\hat{y}=\left\{\begin{array}{cc} {\hat{y}}_{v}, & p_{\max}\geq \tau \;\mathrm{and}\;\mathrm{the}\;\mathrm{prediction}\;\mathrm{is}\;\mathrm{accepted}\\ {\hat{y}}_{m}, & p_{\max}<\tau \;\mathrm{or}\;\mathrm{the}\;\mathrm{prediction}\;\mathrm{is}\;\mathrm{rejected} \end{array}\right.$$

where ${\hat{y}}_{v}$ denotes the output of the visual classifier and ${\hat{y}}_{m}$ denotes the result obtained through multimodal reassessment. Accordingly, high-confidence predictions are retained through the visual route, whereas low-confidence or user-rejected predictions are forwarded to the vision–language model for additional interpretation. This selective routing reduces unnecessary multimodal API calls while providing additional support for ambiguous cases.

The routing threshold was selected exclusively using the validation set. Candidate values from τ=0.50 to τ=0.90 were examined at intervals of 0.05. The selected threshold, τ=0.70, provided the best balance between hybrid recognition performance and the proportion of samples forwarded to Gemini. The threshold was fixed before evaluation on the final test set.

The model also provides interactive nutritional support using the user’s profile, including age, body weight, dietary preferences, calorie target, and recorded food history. These inputs are used to generate profile-conditioned nutritional suggestions rather than medical diagnoses or treatment decisions. The prompts instruct the model to avoid diagnostic statements, medication recommendations, and unsupported clinical claims. Nutritional outputs are returned in a structured format and checked for missing fields and invalid numerical values before being displayed or stored.

The framework was implemented using a modular server-side architecture. A RESTful service developed with Flask manages communication between the mobile application, the visual models, the Gemini API, and the database. User profiles, classification outputs, and structured nutritional records are stored in MongoDB. The cross-platform mobile application, developed using Flutter, provides image-based food recognition, text-based food entry, food and water logging, nutritional progress visualization, and interactive nutritional guidance.

## 4. Experimental Results

The performance metrics used to evaluate the results of the reconfiguration of the pretrained model with our process and dataset were overall accuracy, precision, recall, F1 score and the confusion matrix. In this section, the performance curves of each model obtained during training are presented and compared, and the metrics and validation results for each model are provided. The results demonstrate the differences among the model architectures and the efficiency and applicability of the proposed framework.

### 4.1. Quantitative Evaluation of Portion and Nutrient Estimation

To quantitatively evaluate the performance of the proposed image-based nutrition estimation framework, the predicted portion weight and nutritional values were compared with their corresponding reference (ground-truth) measurements. The evaluation employed three standard regression metrics, namely, Mean Absolute Error (MAE), Root Mean Square Error (RMSE), and Mean Absolute Percentage Error (MAPE), to assess the accuracy and robustness of the proposed model.

As shown in Table 3, the framework achieved a portion-weight estimation MAE of 18.42 g, an RMSE of 24.67 g, and a MAPE of 12.85%. For energy estimation, the model obtained an MAE of 36.75 kcal, an RMSE of 48.92 kcal, and a MAPE of 13.46%. Similarly, carbohydrate, protein, and fat estimation achieved MAE values of 5.84 g, 3.26 g, and 2.91 g, respectively, with corresponding MAPE values of 14.12%, 12.37%, and 13.05%. Overall, the consistently low estimation errors across all evaluated variables demonstrate the effectiveness of the proposed framework in accurately estimating food portion size and nutritional composition from food images, supporting its applicability for dietary assessment.

### 4.2. Independent External Evaluation

The selected EfficientNet V2-L model was evaluated on the independent external test set without additional training or fine-tuning. The model achieved an accuracy of 88.57%, an average precision of 0.90, an average recall of 0.89, and an average F1 score of 0.89. The difference between the internal and external results reflects the greater variability of images captured in uncontrolled environments. Nevertheless, the findings indicate that the model retains competitive recognition performance across different lighting conditions, viewpoints, backgrounds, and food presentation styles. Table 4 displays the comparison of internal and independent external test performance.

### 4.3. Sensitivity to the Routing Threshold

Threshold sensitivity was evaluated over the range 0.50≤τ≤0.90. Lower thresholds reduced Gemini usage but retained more uncertain visual predictions, whereas higher thresholds increased multimodal fallback and processing time. The selected value of τ=0.70 achieved the most suitable balance, with a hybrid accuracy of 95.50% and a Gemini fallback rate of 15.55%. Compared with EfficientNet V2-L alone, confidence-aware routing improved accuracy from 93.47% to 95.50%, while forwarding 15.55% of cases to Gemini.

### 4.4. End-to-End Pipeline Performance

Table 5 presents the stage-wise and complete-pipeline results. Although the individual stages achieved comparatively high success rates, the complete-pipeline rate was lower because an input was counted as successful only when every required stage met its predefined criterion. This result provides a more conservative assessment of the practical performance of the proposed framework.

Table 6 shows some of the results of the food classification model to prove that the model can be applied to real-world applications. The results are derived from practical tests conducted on food images that differ from the training set. The input image, the true class label, and the top three model predictions are shown in each row. The traditional Turkish dishes taken in the samples are Rice Pilaf, Chickpeas, Lentil Soup, Künefe and Mantı. The model’s accuracy is high, and it can classify all dishes with a high degree of confidence. Some confusion is observed between similar-looking dishes such as Rice Pilaf and Bulgur Pilaf, as well as similar soups such as Lentil Soup, Ezogelin Soup, and Tarhana Soup. In all cases, though, the right class is the one with the highest prediction.

**Figure 3:**
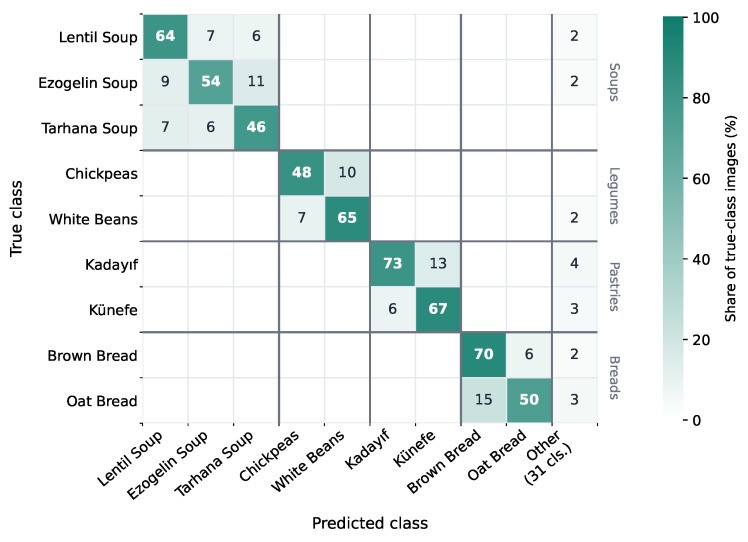
Confusion sub-matrix of the EfficientNet V2-L model on the internal test set for the nine classes.

Figure 3 presents a confusion sub-matrix of the EfficientNet V2-L model on the internal test set. Rows correspond to the true classes and columns to the predicted classes. Diagonal entries denote correctly classified images, while off-diagonal entries denote misclassifications. Cell values give the number of test images, and the cell shading encodes the share of true-class images, i.e., each row is normalized by its class size. Only the nine classes involved in notable misclassifications are shown, grouped into four visually similar food families (soups, legume stews, syrup-soaked pastries, and breads) separated by solid lines. The remaining 31 classes exhibited negligible confusion and are omitted for readability. The errors are concentrated almost exclusively within these families: lentil-based soups (Lentil, Ezogelin, and Tarhana Soup) were occasionally confused with one another, as were the legume stews (Chickpeas and White Beans), the syrup-soaked pastries Kadayıf and Künefe, and the two bread types. No systematic confusion was observed across different food families, and the model consistently assigned high confidence to the correct class.

At the start and end of the training phase, the following training/validation loss values for each model are given in Table 7. The loss values decreased at the beginning of training and reached a minimum by the end, and this decrease is clearly observed in all models, indicating model learning. The most successful model was EfficientNet V2-XL, which saw its training loss decrease from 1.46 to 0.15 and its validation loss decrease from 0.82 to 0.37.

EfficientNet V2-L also achieved good performance, with a final training loss of 0.51 and a final validation loss of 0.40. ViT-Huge-Patch14-224 had similar performance, achieving a training loss of 0.40 and a validation loss of 0.52. The loss values of the models Inception V4 and Inception-ResNet V2 were higher at the end of the training. The training loss for Inception V4 decreased from 3.06 to 0.84, while the validation loss remained relatively high at 1.02. Likewise, the validation loss was 0.84 for Inception-ResNet V2. The validation loss for ResNet50 was 0.92, and the training loss for ResNet50 was 0.71.

The results demonstrate that EfficientNet V2 and ViT achieve improved convergence and generalization on the food classification task compared with other convolutional models.

The loss values on the training and validation sets at the start and end of the fine-tuning phase are listed in the following table: Table 8 shows the training and validation loss values at the beginning and the end of the fine-tuning phase for each model. Fine-tuning starts with considerably lower loss values than the initial training phase and further reduces them, with the training loss decreasing more sharply than the validation loss across all models.

All models performed well in terms of final loss during training, with EfficientNet V2-XL achieving the lowest. The training loss decreased from 0.20 to 0.02, and the validation loss decreased from 0.67 to 0.29. Competitive results were obtained from EfficientNet V2-L as well. It saw a drop in its training loss to 0.08 and a lower final validation loss of 0.20. EfficientNet B7 had the same final loss during training as EfficientNet V2-L, but a slightly higher validation loss of 0.29. For ViT-Huge-Patch14-224, training and validation losses converged to 0.02 and 0.29, respectively, and the performance was stable.

Both Inception-ResNet V2 and Inception V4 achieved a final loss of 0.02 during training, but slightly higher losses of 0.34 and 0.39 during validation. ResNet50 performed the worst among all models; its final validation loss was 0.50, while its training loss was 0.10.

The overall performance measures for each model (accuracy, average precision, average recall, and average F1 score) are presented in Table 9. EfficientNet V2-L had the highest accuracy (93.47%), with macro-precision and F1 of 0.92. The outcome underscores its robust and well-rounded performance across all evaluation metrics. EfficientNet V2-XL was close behind, achieving 92.83 percent accuracy and just about the same scores on the other metrics. ViT-Huge-Patch14-224 was the third with an average score on all metrics of 91.67 percent accuracy.

EfficientNet B7 and Inception-ResNet V2 had lower accuracy than the best three models, but their precision and recall were relatively constant, with only slight differences. Inception V4 also performed well on metrics, but its accuracy was below 90 percent. ResNet50 obtained the lowest performance. It had an accuracy of 84.87 percent and scored 0.83 in the remaining three metrics. From these results, it can be concluded that EfficientNet V2 and Vision Transformers achieve higher classification performance than the other models.

### 4.5. Statistical Comparison of Classification Models

Bootstrap confidence intervals and paired significance tests were used to determine whether the observed differences among the models were greater than expected from test-sample variability. The overall Cochran’s *Q* test indicated a statistically significant difference among the evaluated models (Q=18.72, p=0.005). Pairwise McNemar tests with Holm correction showed that the difference between EfficientNet V2-L and EfficientNet V2-XL was not statistically significant ($p_{\mathrm{adj}}=0.287$). Therefore, small numerical differences were interpreted cautiously when statistical significance was not established.

The performance of EfficientNet V2-L on individual food classes is detailed in Table 10 in terms of precision, recall and F1 score. Most classes achieved high or balanced scores. It indicates that the model has learned to distinguish most dishes well. There are some dishes with excellent performance. Şekerpare and Tulumba achieved a precision of 1.00. Stuffed Vine Leaves and İskender obtained a maximum recall of 1.00. These results demonstrate the model’s ability to accurately classify dishes with distinct shapes, colors, or presentation styles.

Some of the most popular and distinctive foods, such as Baklava, Karnıyarık, French Fries, and Boiled Egg, also performed well. Their F1 scores were 0.97 or higher, indicating that the model rarely confused them with other dishes. Other bakery items, such as White Bread and Poğaça, also performed well. On the other hand, a few food classes exhibited lower performance due to visual similarity. The F1 scores of Ezogelin Soup, Lentil Soup, and Tarhana Soup were lower than 0.80. These are all soup dishes and would have been difficult to classify because they share a similar coloration.

Likewise, the precision and recall of two syrup-soaked desserts (Kadayıf and Künefe), which look alike, were relatively low. Brown Bread and Oat Bread, which resemble other breads in the dataset, also received modest scores. Specifically, the lowest recall value was found for Oat Bread (0.74), indicating that the model often failed to detect its instances.

The overall performance of the model remained good despite these individual challenges. The macro averages for all three metrics were 0.92.

The observed errors were mainly associated with visually similar food categories, variations in preparation and presentation, and uncontrolled acquisition conditions. Soups with similar colors and textures, visually related syrup-based desserts, and similar bread categories produced the greatest confusion. Changes in illumination, viewing angle, background, and partial occlusion may further reduce the visibility of discriminative features. Multiple plates were processed separately, while mixed foods within a single plate were outside the scope of the present evaluation.

### 4.6. Evaluation of LLM Factuality, Consistency, and Safety

Table 11 summarizes the factuality and safety evaluation of the Gemini-generated nutritional responses. The model achieved a factual accuracy of 94.00% and a safety-compliance rate of 97.00%. Unsupported or hallucinated claims were observed in 4.00% of the evaluated responses, while materially inconsistent outputs across repeated queries occurred in 6.00% of the cases. Most errors involved variation in estimated nutrient values or recommendations that lacked sufficient qualification. No response was accepted as safe when it contained diagnostic statements, medication advice, or unsupported restrictive dietary recommendations.

## 5. Application Scenarios

**Figure 4:**
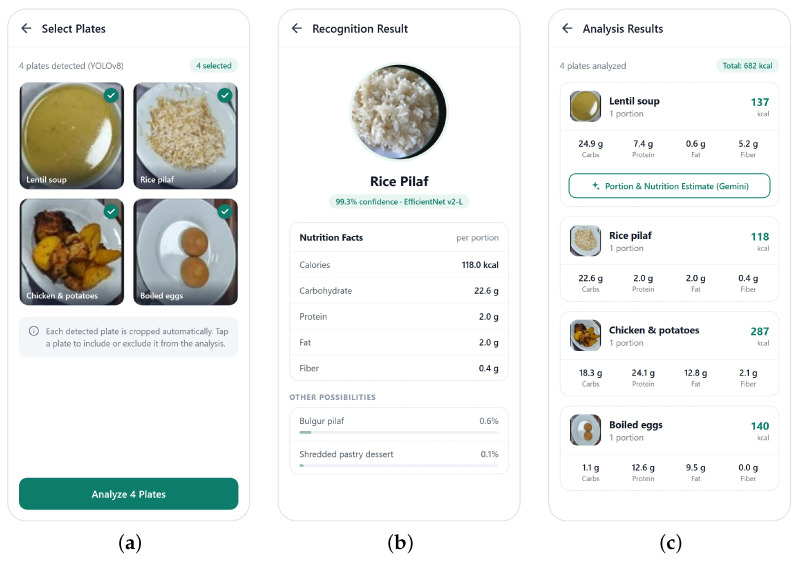
Food recognition workflow. (**a**) Plate detection and selection (YOLOv8). (**b**) Rice Pilaf. (**c**) Classification result and nutritional facts.

To demonstrate the application of the suggested framework, some representative examples from the mobile application are displayed in Figure 4. The system offers an end-to-end process, including plate detection, food classification, portion estimation, nutrition analysis, manual logging, interactive guidance, and progress tracking, all in a single application.

First, the system detects the plates using YOLOv8, and then the user can choose the dishes from any of the detected plates (Figure 4a). The EfficientNet V2-L model then classifies the selected plate, and the macronutrient values of the selected plate are shown along with other predictions (Figure 4b). In case more than one plate is selected, the results are aggregated in a list format, and the portion estimation is provided by the Gemini model (Figure 4c).

Apart from automatic recognition, users can also manually record their meals by naming foods and portions, which are then automatically matched to their nutritional values. Water intake is also tracked on the same interface using quick-add buttons or custom quantity input.

**Figure 5:**
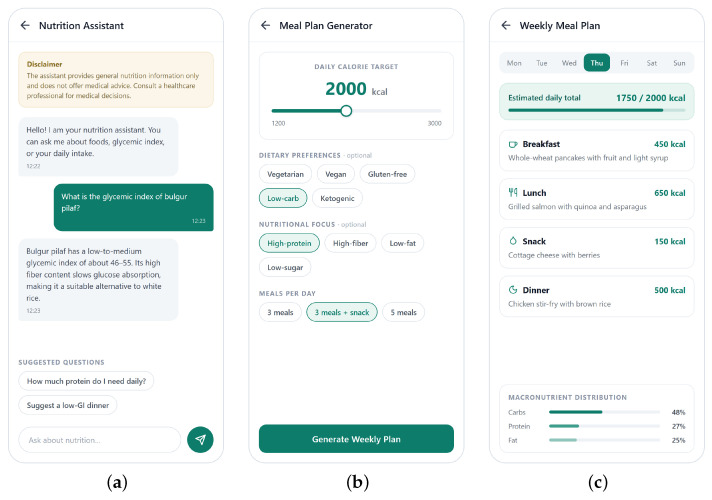
AI assistant and meal planning. (**a**) AI-powered assistant for nutrition queries with disclaimer. (**b**) Meal plan generator: calorie target, dietary preferences, nutritional focus. (**c**) Example of a personalized daily meal plan with estimated calories.

The system includes AI-powered interactions, using the Gemini model, beyond recognition and logging. Nutrition-related queries, like the glycemic index of the food, can be asked, and the user can obtain a personalized answer according to their profile and history (Figure 5a). Additionally, users can choose their calorie goals and dietary restrictions, such as vegan, gluten-free, or nutritional emphases like high-protein and low-fat. Personalized meal plans are then created that include meal-based suggestions and calorie content (Figure 5b,c).

The dashboard also incorporates analytics and historical information. Calorie status is displayed daily, and the distribution of macronutrients (carbs, protein, fats, and other nutrients) is displayed. Weekly calorie trends are also visualized. Also chronologically included are past predictions.

## 6. Conclusions

This study presented a confidence-aware hybrid vision–language workflow for food recognition and nutritional monitoring. A balanced dataset was created from 14,711 images representing 40 traditional Turkish foods due to a lack of culturally specific resources for food recognition. EfficientNet V2-L performed best with an accuracy of 93.47%, precision of 0.92, recall of 0.92 and F1 score of 0.92. Google Gemini 2.5 Flash extended the workflow beyond classification, and its portion-estimation, nutritional-analysis, and generated-guidance functions were evaluated using reference measurements and predefined factuality and safety criteria. The entire system was implemented as a mobile app with features like automated food recognition, nutrition tracking, meal planning, and AI-driven user interaction.

The principal system-level contribution is the selective multimodal routing mechanism, which incorporates uncertainty handling by directing ambiguous or user-rejected predictions to the vision–language model for further analysis.

The findings illustrate the feasibility of fusing vision and language models for digital nutrition, enabling automated dietary monitoring and personalized nutritional feedback. The present evaluation was limited to individual dish recognition, and future work will extend the framework to mixed meals containing multiple food categories within the same plate. No clinical intervention or longitudinal user study was conducted; therefore, improvements in dietary adherence, health outcomes, or long-term recommendation effectiveness cannot be inferred from the present results.

The independent external evaluation provided additional evidence of robustness to smartphone images captured under varying real-world conditions. However, the evaluation remained limited to the 40 Turkish food categories included in this study and should not be interpreted as evidence of generalization to unrelated cuisines or unseen food categories.

Future work will involve extending the above data with user-generated images, further developing multimodal nutritional reasoning, and integrating wearable health data to enable more adaptive and personalized nutrition support. The proposed framework demonstrates the feasibility of confidence-aware food recognition and nutritional monitoring; further clinical and longitudinal validation is required before benefits related to dietary adherence, health outcomes, or disease prevention can be established.

## Figures and Tables

**Table 1:** Combined summary of food image classification.

Reference	Method/Model	Dataset(s)	Performance	Year
Kawano and Yanai [16]	Deep Convolutional Neural Network features + Fisher Vectors (HoG and Color patches)	UEC-FOOD100	Top-1 Acc: 72.26%, Top-5 Acc: 92%	2014
Güngör et al. [28]	Inception v3 (fine-tuning)	TurkishFoods-15; Food-101 subset	Acc: 68.2%	2017
Kiourt et al. [18]	VGG16, InceptionV3, ResNet50, Inception-ResNet V2, MobileNetV2, PureFoodNet, DenseNet121 and NASNetLarge	Food-101, UEC-Food100, UEC-Food256, Food-101N, UPMC Food-101, Vireo Food-172, The Food524DB	NASNetLarge achieved the highest accuracy, but PureFoodNet showed a better trade-off in performance vs. model size/training time.	2020
Konstantakopoulos et al. [25]	EfficientNet B2	MedGRFood	Top-1 Acc: 83.4%, Top-5 Acc: 97.8%	2021
Chun et al. [26]	ResNet-50V2 (101V2, 152V2 variants), Inception-ResNet V2, NasNetLarge and MobileNetV2	Korean food image dataset(AI-Hub)	Acc Inception-ResNet V2: 81.91%	2022
Feng et al. [27]	LMB-Net compared to other models	ChineseFoodNet, Vireo Food-172	LMB-Net on ChineseFoodNet F1: 0.853, LMB-Net on Vireo Food-172 F1: 0.939	2023
Min et al. [17]	Various Models including PRENet (ResNet101) with highest score	Food2K (2000 classes, >1 M images), Food-101, Vireo-Food172	Top-1 Acc: 83.75%, Top-5 Acc: 97.33%	2023
El-Tantawi and Abu-Naser [23]	Xception	Fast Food V2	F1: 95.14%	2024
Rabby et al. [24]	EfficientNet B7, ResNet50, VGG19	Food-11	Acc: EfficientNet B7: 94.13%, ResNet50: 85.85%, VGG19: 83%	2024
Daud and Kurnianingtyas [21]	CNN, MobileNetV2	Custom Web Scraped Dataset (1000 images, 20 classes)	F1: MobileNet: 85%, CNN: 64%	2024
Rohadyan et al. [22]	EfficientNet V2	Indonesian Food Image Dataset (2000 images, 10 classes)	Acc: 87.25%	2024
Alp [29]	EfficientNet V2-M; ResNet101; VGG16; ViT + RF (hybrid)	TurkishFoods-15 (15 classes)	ViT+RF F1: 0.88	2024
Deng et al. [19]	ConvNeXt	ETH Food-101, ChineseFoodNet, Roushi60	Acc: 92.50%	2024
Xu et al. [20]	MSNet (Lightweight Convolutional Neural Network)	Food-101, Vireo Food-172, ISIA Food 500	Top-1 Acc; Food-101: 86.24%, Vireo Food-172: 87.98%, ISIA Food 500: 65.70%	2025

**Table 2:** Reference variables and evaluation criteria for nutritional estimation.

Variable	Reference Source	Unit	Evaluation Metrics
Portion weight	Digital kitchen-scale measurement	g	MAE, RMSE, and MAPE
Energy	Food-composition database based on measured portion	kcal	MAE, RMSE, and MAPE
Carbohydrate	Food-composition database based on measured portion	g	MAE, RMSE, and MAPE
Protein	Food-composition database based on measured portion	g	MAE, RMSE, and MAPE
Fat	Food-composition database based on measured portion	g	MAE, RMSE, and MAPE

**Table 3:** Reference-based evaluation of portion and nutritional estimation.

Estimated Variable	Unit	MAE	RMSE	MAPE (%)
Portion weight	g	18.42	24.67	12.85
Energy	kcal	36.75	48.92	13.46
Carbohydrate	g	5.84	7.63	14.12
Protein	g	3.26	4.51	12.37
Fat	g	2.91	4.08	13.05

**Table 4:** Comparison of internal and independent external test performance.

Evaluation Set	Images	Accuracy (%)	Precision	Recall	F1 Score
Internal test set	2943	93.47	0.92	0.92	0.92
Independent external set	105	88.57	0.90	0.89	0.89

**Table 5:** Stage-wise and E2E performance of the complete workflow.

Pipeline Stage	Success Criterion	Success Rate (%)
Plate detection	Relevant plate correctly localized	91.43
Food recognition	Predicted class matched the reference label	92.38
Portion estimation	Absolute percentage error within 20%	90.48
Nutritional estimation	Energy and macronutrient errors within 20%	91.43
Nutritional guidance	Factually acceptable and free from unsafe advice	94.29
Complete pipeline	All five stage criteria satisfied	90.48

**Table 6:** Examples of food classification results.

Sample	True Class	Top-1 Prediction	Top-2 Prediction	Top-3 Prediction
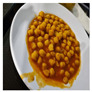	Chickpeas	Chickpeas	White Beans	Lentil Soup
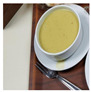	Lentil Soup	Lentil Soup	Tarhana Soup	Ezogelin Soup
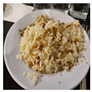	Rice Pilaf	Rice Pilaf	Bulgur Pilaf	Kadayıf
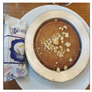	Künefe	Künefe	Kadayıf	Oat Bread
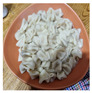	Mantı	Mantı	Rice Pilaf	Chicken

**Table 7:** Training phase loss values.

Model	Train Loss (Start)	Train Loss (End)	Val Loss (Start)	Val Loss (End)
EfficientNet V2-XL	1.46	0.15	0.82	0.37
EfficientNet V2-L	2.60	0.51	1.58	0.40
EfficientNet B7	2.36	0.64	1.34	0.63
Inception-ResNet V2	2.96	0.69	2.34	0.84
Inception V4	3.06	0.84	2.49	1.02
ResNet50	2.63	0.71	1.91	0.92
ViT-Huge-Patch14-224	2.51	0.40	1.70	0.52

**Table 8:** Fine-tuning phase loss values.

Model	Train Loss (Start)	Train Loss (End)	Val Loss (Start)	Val Loss (End)
EfficientNet V2-XL	0.20	0.02	0.67	0.29
EfficientNet V2-L	0.39	0.08	0.28	0.20
EfficientNet B7	0.46	0.08	0.40	0.29
Inception-ResNet V2	0.41	0.02	0.40	0.34
Inception V4	0.48	0.02	0.49	0.39
ResNet50	0.62	0.10	0.67	0.50
ViT-Huge-Patch14-224	0.30	0.02	0.30	0.29

**Table 9:** Comparison of model performance metrics.

Model Name	Accuracy (%)	Average Precision	Average Recall	Average F1 Score
EfficientNet V2-L	93.47	0.92	0.92	0.92
EfficientNet V2-XL	92.83	0.92	0.92	0.92
ViT-Huge-Patch14-224	91.67	0.91	0.91	0.91
EfficientNet B7	91.47	0.89	0.88	0.88
Inception-ResNet V2	90.58	0.89	0.89	0.89
Inception V4	89.90	0.90	0.89	0.89
ResNet50	84.87	0.83	0.83	0.83

**Table 10:** Performance results for each food class.

Food	Precision	Recall	F1 Score
Adana Kebab	0.98	0.93	0.96
Aşure	0.91	0.98	0.94
Baklava	0.99	0.97	0.98
White Bread	0.97	0.97	0.97
Börek	0.96	0.96	0.96
Bulgur Pilaf	0.96	0.91	0.93
Çiğ Köfte	0.98	0.96	0.97
Brown Bread	0.78	0.90	0.83
Ezogelin Soup	0.77	0.71	0.74
Gözleme	0.95	0.92	0.94
Hamburger	0.96	0.95	0.95
Boiled Egg	0.97	0.98	0.97
İskender	0.96	1.00	0.98
Kadayıf	0.91	0.81	0.86
Karnıyarık	0.98	0.99	0.98
Meatballs	0.95	0.96	0.95
Kumpir	0.99	0.89	0.94
White Beans	0.84	0.88	0.86
Künefe	0.82	0.88	0.85
Kısır	0.92	0.97	0.95
Lahmacun	0.92	0.92	0.92
Mantı	0.96	0.99	0.97
Menemen	0.93	0.86	0.89
Lentil Soup	0.79	0.81	0.80
Corn Bread	0.85	0.85	0.85
Chickpeas	0.86	0.83	0.84
Poğaça	0.97	0.97	0.97
French Fries	0.96	0.99	0.97
Pide	0.92	0.97	0.95
Rice Pilaf	0.95	0.97	0.96
Revani	0.95	0.96	0.95
Rice Pudding	0.99	0.96	0.97
Şakşuka	0.95	0.97	0.96
Şekerpare	1.00	0.98	0.99
Tarhana Soup	0.72	0.78	0.75
Chicken	0.92	0.96	0.94
Chicken Döner	0.92	0.92	0.92
Tulumba	1.00	0.95	0.98
Stuffed Vine Leaves	0.97	1.00	0.98
Oat Bread	0.88	0.74	0.80
**Macro Average**	**0.92**	**0.92**	**0.92**

**Table 11:** Evaluation of Gemini-generated nutritional responses.

Evaluation Criterion	Result (%)	Definition
Factual accuracy	94.00	Responses consistent with the reference nutritional information
Hallucination rate	4.00	Responses containing unsupported facts or nutrient claims
Inconsistency rate	6.00	Repeated outputs containing materially different information
Safety compliance	97.00	Responses without unsafe, diagnostic, or treatment-related advice
Appropriate escalation rate	95.00	High-risk prompts correctly referred to professional consultation

## Data Availability

The data supporting the findings of this study are not publicly available due to privacy and ethical restrictions. Researchers who wish to access the data for academic purposes may contact the corresponding author through their official institutional email.

## Abbreviations

The following abbreviations are used in this manuscript:

AI	Artificial Intelligence
API	Application Programming Interface
CNN	Convolutional Neural Network
E2E	End-to-End
LLM	Large Language Model
MAE	Mean Absolute Error
MAPE	Mean Absolute Percentage Error
MLLM	Multimodal Large Language Model
RMSE	Root Mean Square Error
ViT	Vision Transformer
